# Beyond daily totals: meal-level digestible indispensable amino acid score reveals how food groups shape protein quality in vegan diets

**DOI:** 10.3389/fnut.2026.1752697

**Published:** 2026-02-12

**Authors:** Bi Xue Patricia Soh, Matthieu Vignes, Nick W. Smith, Pamela R. von Hurst, Warren C. McNabb

**Affiliations:** 1 Sustainable Nutrition Initiative, Riddet Institute, Massey University, Palmerston North, PMN, New Zealand; 2 School of Mathematical and Computational Sciences, Massey University, Palmerston North, PMN, New Zealand; 3 The New Zealand Institute for Bioeconomy Science Ltd. (Plant and Food Group), Palmerston North, PMN, New Zealand; 4 School of Sport, Exercise and Nutrition, College of Health, Massey University, Auckland, AUC, New Zealand

**Keywords:** amino acids, cluster analysis, protein intake, protein quality, vegan diets, vegan meals

## Abstract

**Introduction:**

Vegan diets rely solely on plant-based proteins, which often supply fewer digestible indispensable amino acids (IAAs) than omnivorous diets. Low protein quality can leave IAAs undersupplied even if the protein intake meets the recommended intakes. Mixtures of plant-based foods in meals can complement limiting IAAs and improve the synchronized delivery of all IAAs for optimal metabolic function. Assessing how food group compositions differ among meals of varied protein quality is essential to determine the ideal proportions of plant-based foods to improve the Digestible Indispensable Amino Acid Score (DIAAS) of vegan meals.

**Methods:**

The DIAAS was calculated for each meal found in the 4-day food diaries of 193 New Zealand vegans. Principal component analysis and k-means clustering were applied to identify food groups associated with meals of varying DIAAS, and meal level clusters were compared to participants’ daily protein intake.

**Results:**

Eight distinct meal clusters emerged. Lower-DIAAS meals were characterized by higher quantities of fruit, grain foods, potatoes, and sugary condiments with significantly lower (*p* < 0.001) weight share of legumes than higher-DIAAS meals, which had a ~ 2:1 legume: grain profile. IAA contribution differed across plant-based food groups as grains, nuts, and seeds supplied more sulfur-amino acids while legumes and extruded plant protein isolates (such as protein powders) supplied more lysine. Notably, lower protein quality meals appeared most often in daily patterns with lower total protein and IAA intake, but also occurred when daily protein was high, underscoring that day-level protein adequacy can mask meal-level deficits.

**Discussion:**

Deliberate meal-level complementation of plant-based foods is necessary to ensure protein quality in vegan dietary patterns.

## 1 Introduction

Interest in vegan diets is rising within nutrition and public health due to their environmental sustainability potential, ethical considerations regarding animal welfare, and potential health benefits (1, 2). However, a parallel body of literature has emerged to highlight the risks of nutrient deficiencies of vegan diets, when nutrient-dense and highly bioavailable animal-sourced foods are entirely replaced with plant-based (PB) alternatives—particularly if the diet is poorly planned or relies on a limited variety of foods (3). Hence, while the motivations to adopt such diets may be compelling for some individuals, ensuring that fully plant-based diets meet nutrient requirements demands close examination.

The nine indispensable amino acids (IAAs) may be at risk of deficiencies in a poorly planned vegan diet and are thus the nutrients of interest in this study. The IAAs must be sufficiently provided through dietary protein, owing to the body’s inability to synthesize them endogenously (4). Plant sources generally have inferior protein quality compared to animal-sourced foods—a concept encompassing the content of digestible and utilizable/bioavailable IAAs to match the body’s requirements (5, 6).

It takes knowledge and intention, but gaps in the IAA profile of vegan meals can be fulfilled through the combinations of diverse plant foods (7). This may be more practical at the meal level, where different foods are mixed and consumed in the same eating occasion (EO). Even so, achieving high protein quality in vegan meals is further challenged by the lower digestibility of plant proteins—as compared to animal proteins—due to the presence of anti-nutritional factors (ANFs) such as phytates, dietary fiber, and enzyme inhibitors in traditional and minimally processed plant foods (8–10). These ANFs limit the postprandial metabolic availability of IAAs in the body (11). As described in our past review, while certain processing mechanisms, including heat treatments, may reduce the presence of ANFs, this varies across different foods and types of ANF (12). Therefore, when evaluating the protein quality of vegan meals, the extent of IAA digestibility in the human body must be properly accounted for to avoid overestimating protein adequacy in vegan diets (13).

The digestible indispensable amino acid score (DIAAS) is currently the recommended metric for protein quality (14). While DIAAS is designed to indicate the protein quality of single foods, it can be applied in the context of meals due to the additive nature of the underlying true ileal digestibility (TID) of IAA values for various foods in a meal (15). A DIAAS of at least 100% indicates that 1 g of the protein from the meal contains at least the quantity of IAA present in 1 g of the reference IAA pattern (15, 16). This provides a straightforward way to identify high-protein quality vegan meals and subsequently evaluate the proportions of PB foods needed to achieve highly utilizable protein. The relative quantities of component foods in high-protein quality meals can then be adjusted to meet an individual’s specific protein and IAA requirements.

The overall aim of this study was to analyze the protein quality of vegan diets at the meal level, using real-world meal consumption data provided by New Zealand (NZ) vegans. By comparing how food group compositions differ among meals of varied protein quality, we can evaluate the ideal proportions of plant-based food sources needed to derive meals of higher protein quality. When protein adequacy is only assessed by comparing the total protein intake of the day with daily requirements, protein distribution across meals of the day and the protein quality of individual meals are not considered. The concept of protein quality encompasses the provision of IAAs from foods that can be available to the body’s metabolic processes. Considering the potential implications of dietary protein distribution over time on body composition and metabolism (17, 18), determining protein quality at both daily and meal levels is necessary. Thus, our secondary aim was to quantify the association between meal-level protein quality and daily protein adequacy by estimating the probability that meals with high protein quality are observed in days meeting protein targets.

## 2 Methods

### 2.1 Study design and data collection

Meals for analysis were obtained from the Vegan Health Research Program, a cross-sectional study which examined the associations between vegan dietary patterns and nutrition and health outcomes among NZ vegans. Through convenience sampling, a total of 193 healthy individuals who have followed a vegan diet for at least 2 years were recruited. Ethical approval was granted by the Health and Disability Ethics Committee (HDEC 2022 EXP 12312), and written consent was obtained from all participants before data collection.

The full demographic characteristics of the study population can be found in our past study (13). Briefly, 52 men and 141 women participated in the study, and New Zealanders of European descent constituted the majority of the population. While the BMI was within the normal range for both sexes, women had a higher percentage of body fat as compared to men. Women as compared to men consumed lower energy and total protein intake per day as compared to men.

A four-day food diary that detailed each individual’s food and beverage consumption was submitted. Foods with the same date and time stamps were assumed to be consumed together and were categorized into one EO. Daily records containing food items with missing time stamps were excluded. A total of 766 daily dietary records were included in the analysis. Single foods that were not consumed as part of a meal and have low digestible protein (<1 g/100 g of food) were excluded from the analysis. This mostly included beverages (coffees, teas, alcoholic beverages, juices, PB beverages) and fruits. A total of 603 single foods with mostly low protein content (<1 g/100 g of food) were removed from a list of 4,345 possible meal combinations.

### 2.2 Data processing

Using the FoodWorks Professional nutrient analysis software (Xyris, Brisbane, Australia, 2022), all consumed items in the diaries, as well as individual ingredients in recipes, were matched to crude protein content, using the nitrogen-to-protein conversion factor in the NZ Food Composition Database (NZFCD) (19). As IAA compositions were absent from the NZFCD, items were matched to the United States Department of Agriculture (USDA) food composition data (20) and then normalized to the protein content of foods from NZ to approximate the IAA quantity in NZ foods. Food matching guidelines from the FAO/INFOODS were followed (21) and when the exact match was unattainable, the food item was matched to several possible food items, and the average value for protein and IAA was calculated.

An estimated digestibility coefficient of between 0 and 1 for total protein and IAAs when available for each specific food item (both traditional and novel PB products) in the food diaries was obtained from available literature at the time of writing (22–25). For example, beans and peas (the legume FG) had a TID value of 0.76 and 0.78, respectively. The TID values of each food group were then multiplied by the content of total protein and each IAA to provide a more accurate approximation of the quantity of each nutrient that can be utilized for metabolic functions in the human body, and for the subsequent calculation of DIAAS. When food items were a result of cooked or store-bought recipes, each ingredient also had adjustments made to its TID value, and the digestible quantities of each nutrient were totaled for each recipe.

TID adjustments were applied directly for total protein, tryptophan, threonine, leucine, lysine, methionine, cystine, and histidine. Digestibility adjustments were made while the sulfur amino acids (SAAs) were derived by adding methionine and cystine after adjustment. At the time of writing, data limitations for phenylaniline, tyrosine, isoleucine, and valine, TID-adjustments could not be applied to these IAAs, and consequently, DIAAS calculations were not determined with the inclusion of these three IAAs.

Each food item in the diaries was then categorized into a broader food group (FG). FG classification was guided by the 2008/2009 NZ Adult Nutrition Survey (26). For example, wheat products such as pasta, noodles, cereals, and bread, as well as grain-based beverages, were classified under “grains and pasta.” Beans, peas, pulses such as lentils, and soy products fall under “legumes and pulses.” Protein isolates and concentrates that were consumed as powdered beverages were classified as “isolates.” Novel food products were matched to the FG of the principal protein-contributing ingredients (27), often pea, soy, or textured vegetable protein.

### 2.3 Data analysis

#### 2.3.1 Protein quality of meals

In this study, we refer to the mixed meals as the combination of all foods and beverages consumed in the same EO. The TID-adjusted IAA content was calculated and expressed as mg of AA per gram of dietary crude protein per meal (28, 29). This value was then divided by the same dietary IAA in 1 g of the reference protein, also referred to as the AA scoring pattern (Table 1)—the ideal IAA composition required to meet the body’s requirements (30). Considering that the baseline vegan diets were obtained from adult participants, aged 18 years and above, this study utilizes the AA scoring pattern for adults (>18 years of age) (28).

**Table 1:** Reference patterns of IAAs, expressed as mg per 1 g of food protein.

IAA	His	Leu	Lys	SAA	Thr	Tryp
References (mg/g of food protein)	15	59	45	22	23	6

IAA, indispensable amino acid; His, histidine; Leu, leucine; Lys, lysine; SAA, sulfur-amino acids; Thr, threonine; Tryp, tryptophan.

The lowest value of the digestible IAA reference ratio thus represents the most limiting IAA in the mixed meal and denotes the DIAAS. We illustrate the above-described calculation using an example of a vegan mixed meal consumed by one participant in Table 2. Each meal component was categorized into its respective FG. For example, the “gravy,” which is primarily made up of cashew nuts, was classified as “nuts and seeds.” Meal weight (g), crude protein (g), TID-adjusted protein (g), and IAA (mg) contents were listed. Digestible IAA reference ratio was expressed as digestible IAA in 1 g protein of the mixed diet/mg of the same dietary IAA in 1 g of the reference protein (Table 1) (28). In this example, the food items, “gravy” and “meatballs,” were the result of a home-cooked recipe, and the TID-adjusted values reflected in Table 2 were a summation of the nutrient content of each recipe. “White rice” existed as a single food item (not part of a recipe but rather, consumed on its own), with a TID value of 0.9 that was multiplied by the crude protein value to provide a TID-adjusted protein value of 0.81 g.

**Table 2:** Sample calculation of one baseline vegan mixed meal with leucine as the most limiting IAA.

Food	FG	Weight in meal (g)	Crude protein in meal (g)	TID-adjusted protein (g)	TID—adjusted AA content (mg) for each food in the mixed meal								Total
					Tryp	Thr	Leu	Lys	Met	Cys	SAA*	His	
Gravy	Nuts and seeds	17.3	2.08	1.58	24.0	61.3	129.4	82.2	34.7	36.5	71.1	41.2	
“Meatballs”	Legumes and pulses	92.6	12.6	8.08	120.2	312.1	573.3	488.3	100.5	113.9	214.5	209.9	
White rice	Grains and pasta	29.0	0.90	0.81	9.41	26.3	60.3	29.9	18.2	18.0	36.1	20.9	
Totals													
		138.9	15.6	10.5	153.6	399.7	762.9	600.5	153.4	168.4	321.7	272.1	
					Digestible IAA reference ratio								DIAAS
					1.64	1.11	0.83	0.86	*	*	0.94	1.16	83% (Leu)
				Weight proportion of each FG to nutrient contribution									
Gravy	Nuts and seeds		0.13	0.15	0.15	0.15	0.17	0.14	0.23	0.23	0.22	0.15	
“Meatballs”	Legumes and pulses		0.80	0.77	0.78	0.78	0.75	0.81	0.66	0.68	0.67	0.77	
White rice	Grains and pasta		0.06	0.08	0.06	0.07	0.08	0.05	0.12	0.11	0.11	0.08	

FG, Food group; DIAAS, Digestible Indispensable Amino Acid Score; TID, True-ileal digestibility; Tryp, Tryptophan; Thr, Threonine; Leu, Leucine; Lys, Lysine; Met, Methionine; Cys, Cystine; SAA, Sulfur-amino acids; His, Histidine *SAA = met + cys. TID-adjusted values for the aromatic amino acids (phenylalanine and tyrosine), isoleucine, and valine are not calculated due to insufficient TID data for these IAAs. Example of weight proportion of TID-adjusted tryptophan from “gravy” is computed by taking 24.0 mg divided by 153.6 mg.

#### 2.3.2 Meal component analysis

Baseline mixed meals were classified into two strata based on their DIAAS scores: higher protein quality (DIAAS ≥75%) and lower protein quality (DIAAS <75%) (28, 29). We acknowledge that the precise rationale for this cut-off is not fully established and could vary across different food matrices (31, 32). This cut-off, however, provides a pragmatic approach in this study to categorize meals to different levels of protein quality for systematic comparison and subsequent modification of meals to increase DIAAS.

Meal compositions within each DIAAS stratum were evaluated with the following steps. The proportional contribution of each contributing FG to protein and IAA within a mixed meal was computed as the ratio of the nutrient quantity of each FG to the total nutrient quantity of the mixed meal. Specifically, protein contribution was expressed as grams of protein provided by each FG divided by the total protein (g) of the meal. The same approach was applied to each IAA, where the IAA quantity (mg) provided by each FG was divided by the total IAA of the meal. Contributions thus summed to 1 across FGs within each mixed meal for each nutrient. Table 2 provides a sample of the calculated weight proportion. To summarize across all meals, the mean contribution to each nutrient for each DIAAS stratum was computed and displayed as a continuous scale using a heat map.

The variation in FG weight (g) contributions when present in meals was explored with ridgeline density plots, which illustrated the frequency of a given FG as a proportion of each meal’s weight for each DIAAS stratum. The Wilcoxon rank-sum test was used as the non-parametric test to compare the relative contributions of each FG between higher and lower DIAAS meals. FG shares were expressed as ratios (e.g., legumes: grains, legumes: nuts/seeds; grains: nuts/seeds) to provide a quantitative estimation of the balance required among FGs to achieve higher protein quality meals.

#### 2.3.3 Meal pattern analysis with principal component analysis and k-means clustering

Each mixed meal was expressed as the proportional contribution of FGs to total meal weight. To reduce sparsity and improve interpretability, minor groups such as beverages, yeast and other condiments, and sugars and sweet that often had no weight share were collapsed into a single category, “others.” As the food-group data were compositional (all shares of the FGs sum to one), PCA was performed on data that had undergone centered log-ratio (CLR) transformation (33). The two principal components (PC1 and PC2), which explained the largest proportion of variance in the meal compositions, were retained for visualization. Each meal was plotted in a two-dimensional map for further interpretation (34, 35). FGs with large positive PC1 loadings drive points to the right of the plot, while negative loadings position corresponding points to the left. Likewise, large positive PC2 loadings push points upward and strong negative loadings pull points downward. Meals with very low DIAAS <10 were excluded from the final analysis. Inspection of these meals indicated that extremely low DIAAS values were driven by the absence or near-absence of one or more IAAs. For some foods, tryptophan was not recorded for some foods, resulting in a DIAAS of 0. There were also instances where meals provided negligible protein or IAA content. Nevertheless, to assess the impact of this exclusion, PCA was rerun to include all meals, with no notable shifts in PC loadings for all FGs, apart from slight shifts in “legumes and pulses” and “others” FGs as a result of soy milk and other beverages, respectively. An isolated cluster of meals with DIAAS equating to 0 was found.

To explore the clustering of meals according to their food-group profiles, k-means clustering was applied to the PCA scores, and the average silhouette width was tabulated for candidate values of *k* = 2–15. The value of k with the highest average silhouette width provides guidance to derive the number of clusters in which each meal fits in comparison to neighboring clusters. Domain knowledge was also utilized to determine the optimal k value. Cluster assignments were then visualized in the PCA space and differentiated by point shapes and color gradient, which represented DIAAS. Cluster centroids were calculated to approximate the mean protein quality of meals, as represented by DIAAS of each cluster.

#### 2.3.4 Linking meal pattern analysis to daily diets

Previously, we performed cluster analysis of daily protein intake patterns and categorized individuals according to the distribution of protein and IAA intake across EOs of the day (36). In another study, we also identified daily diets where participants had at least one shortfall either in protein or any IAA, as compared to their daily requirements (37). These analyses, however, did not consider the composition of each meal or EO. With PCA and k-means clustering in this study, distinct FG patterns were identified at the meal level and related back to the findings from our past research. This aimed to determine the probability of consuming a high-protein-quality meal among individuals in clusters with higher average per-EO protein intake and no daily protein or IAA shortfalls.

Each meal was assigned back to the daily temporal cluster from which it originated—the cluster assigned to that participant’s meals for that day (36). These are termed as dietary clusters with cluster 1 containing participants whose mean intake of protein and IAA per EO was the lowest, cluster 3 containing participants whose mean intake of these nutrients per EO was the highest, and cluster 2 was an intermediate cluster. Our previous research had not examined meal compositions for protein quality, and the current study therefore aims to address this. The probability that a meal from a PCA-derived meal cluster falls into each pre-assigned daily cluster was calculated. A similar probabilistic approach was also applied to test whether specific meal compositions were more common among individuals who exhibited no nutrient shortfalls (37). A chi-square test of independence was applied to determine the association between PCA-derived meal clusters and specific daily dietary patterns.

## 3 Results

### 3.1 Meal component analysis

**Figure 1:**
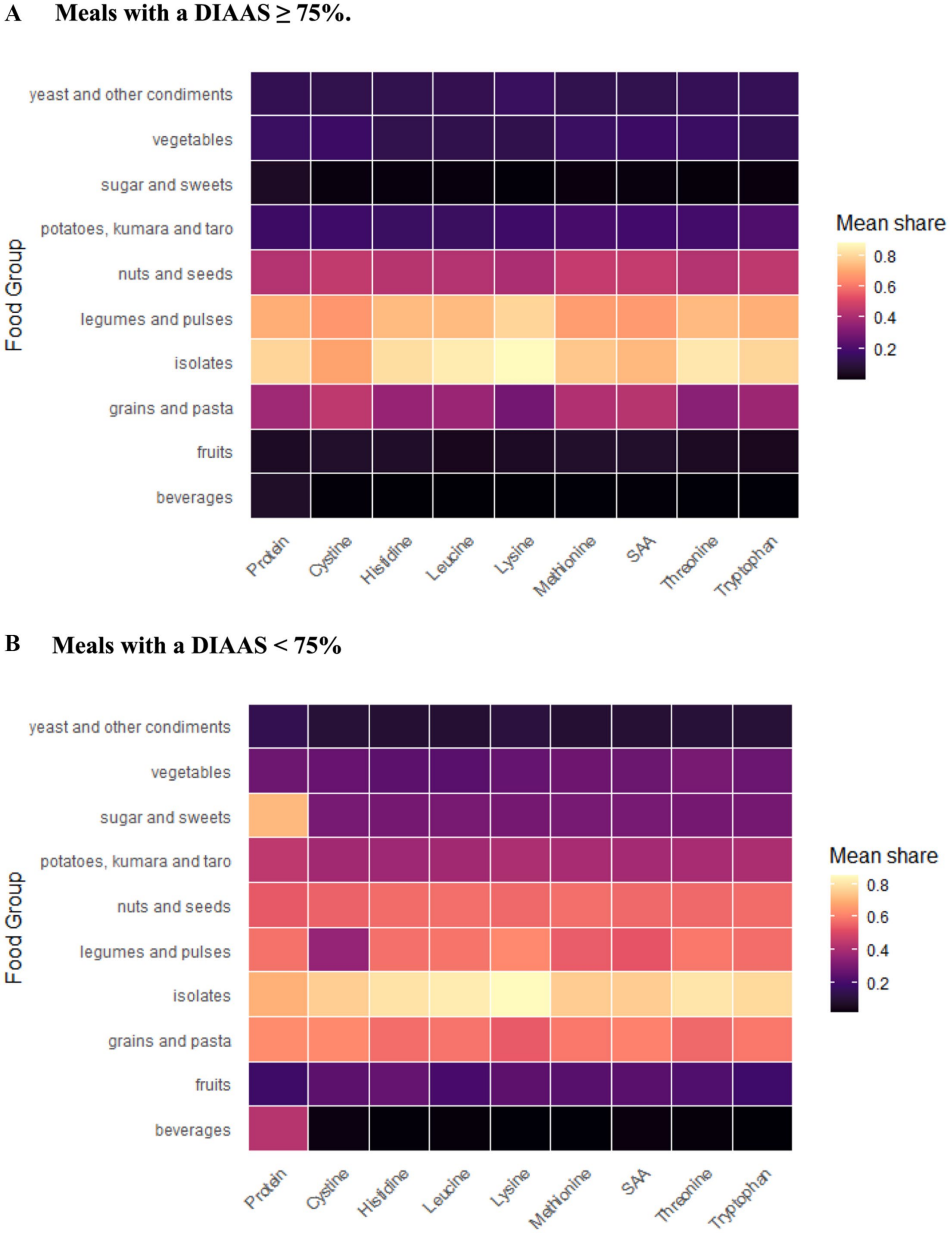
Heat map showing the mean share of protein and IAA content contributed by each FG when present in meals. Lighter colors show a larger share. Meals with higher protein quality (DIAAS ≥75%) are characterized by a higher contribution of protein and IAA from isolates, legumes/pulses than from other FGs **(A)**, as compared to meals with lower protein quality (DIAAS <75%) where several FGs contribute more equally to total protein and IAAs **(B)**.

The meals in this vegan cohort were predominantly lower in protein quality—822 meals had a DIAAS ≥75%, and 2,920 meals <75%. The mean contribution of FGs to total nutrient content when present in meals with higher (DIAAS ≥75%) and lower protein quality (DIAAS <75%) is shown in separate heat maps (Figure 1). Lysine was the most frequently limiting IAA in meals (56.6% of meals), followed by SAAs (21%), leucine (13.4%), and tryptophan (8.7%). Histidine and threonine were rarely limiting.

In meals with higher DIAAS, the largest contributions to total protein and overall protein quality were from isolates, legumes, and pulses, with ≥0.6 mean share of protein and each IAA. Lower mean shares were observed from nuts and seeds, and grains and pasta (approximately 0.2–0.5) (Figure 1A). In particular, lysine was mostly contributed by isolates, and legumes and pulses, in contrast to the other FGs. Conversely, grain foods contributed less to lysine as compared to their contribution to other IAAs but provided a larger share of the SAAs. Grain foods were more dominant in meals of lower than higher protein quality, where they contributed more toward protein and IAA. As expected, sugar and sweets, fruits and beverages did not contribute meaningfully to protein content or quality. In particular, sugar and sweets, and beverages, respectively, formed <0.1 mean share of protein in higher DIAAS meal scores, in contrast to 0.7 and 0.4 in lower DIAAS meals (Figure 1B). The contribution of isolates to the nutrient density was fairly similar in both strata. As indicated by the mean share or color gradient of both heat maps in Figure 1, legumes, pulses, and isolates were clearly dominant FGs providing protein and IAAs in higher DIAAS meals. Conversely, the provision of these nutrients was more equally distributed across FGs in lower DIAAS meals.

**Figure 2:**
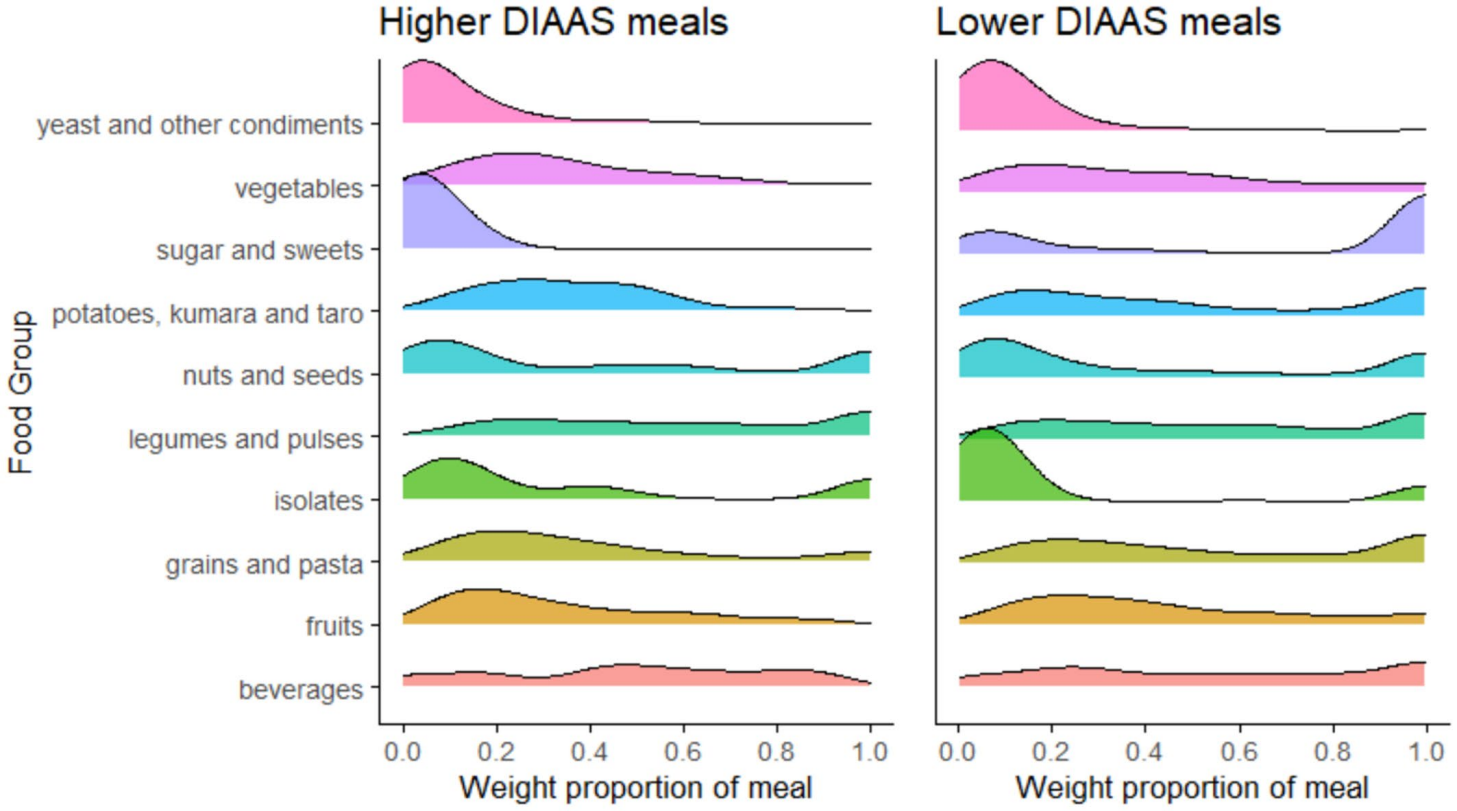
Ridgeline plots showing the distribution of food-group weight shares (grams in meals) when present in the meals in higher and lower protein quality strata. The x-axis represents the proportion of the meal’s weight contributed by each FG. The height of the ridge (y-axis) reflects the relative frequency of meals. Taller ridges show a higher concentration of meals at the particular weight proportion, and flatter ridges show a wider spread of meals across different weight proportions.

Food group contributions by weight were analyzed with two complementary approaches. Within each DIAAS stratum, the distribution of each FG to the weight proportion of a meal is visualized with ridgeline plots in Figure 2, which shows higher weight proportions for a few FGs in the lower DIAAS than higher DIAAS meals. In this case, the distributions of within-meal FG weight proportions were examined conditional on the presence of the FG. For example, for sugar and sweets, the curve for the higher DIAAS meals peaked close to zero, indicating that these foods were minimal or absent in a large share of higher protein-quality meals, in direct contrast to the curve for lower protein-quality meals. For potatoes, kumara, and taro, and grains and pasta, peaks were observed at 0.9 in the lower DIAAS meals but not in the higher DIAAS meals. In the higher DIAAS meals, the curves for these FGs peaked between 0.0 to 0.6. Interestingly, the distribution of the more protein-dense FGs—legumes and pulses, and nuts and seeds—showed less distinction between higher and lower DIAAS meals, indicating that the quantity, when present in meals, did not vary systematically with DIAAS.

**Figure 3:**
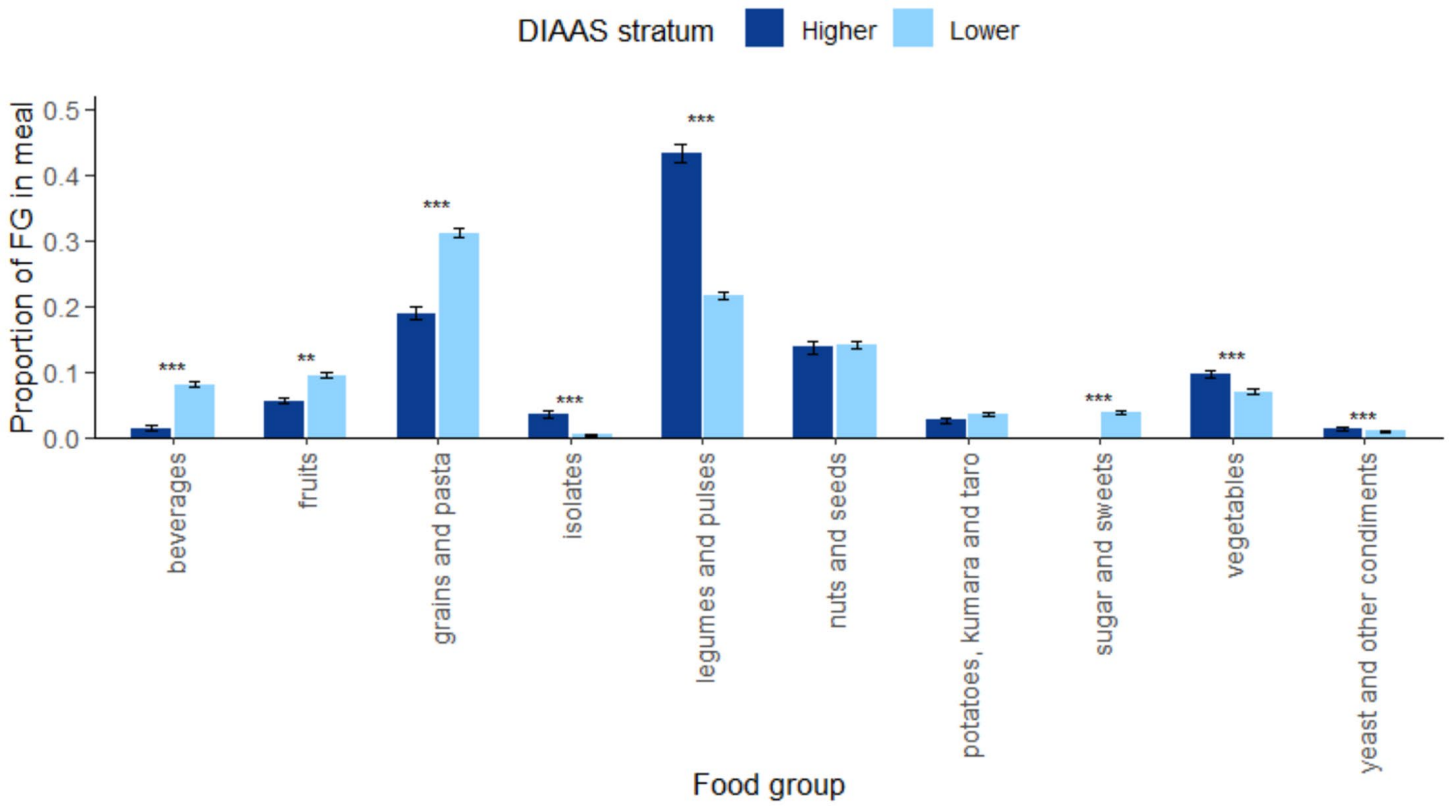
Bar plots comparing the mean meal proportion in % contributed by each FG between higher and lower DIAAS stratum, with error bars representing standard error, with ** representing *p* < 0.01 and *** representing *p* < 0.001. Proportions of FGs accounted for their weight contribution and prevalence in the meals of each DIAAS stratum and sum to 1.

Bar plots (Figure 3) showed differences in FG contributions between higher and lower-protein quality meals when FG prevalence in meals (how often a FG appears in each meal) was accounted for with the weight contribution of each FG. While results in Figure 2 did not consider prevalence, the step here computed the mean FG share by averaging weight proportions across all meals, where absent FGs in meals were assigned a value of zero. This approach thus incorporated FG-prevalence, defined here as the frequency at which an FG appears in meals. This distinguishes FGs that are frequently used in small quantities from those that could be used more infrequently but in larger quantities. Wilcoxon rank-sum test showed significant differences in the mean proportion of FG contribution between higher and lower DIAAS stratums for all FGs, except nuts and seeds (*p =* 0.622) and potatoes, kumara, and taro (*p =* 0.699).

When prevalence was not considered, legumes and pulses had a similar weight proportion of approximately 0.6 in higher DIAAS meals and lower DIAAS meals, as indicated in Figure 2, indicating comparable portion sizes in meals from different DIAAS strata. However, the prevalence of legumes and pulses appearing as a component in higher DIAAS meals was 0.7 as compared to 0.4 in lower DIAAS meals, hence explaining the significant difference observed in Figure 3. This reflects more frequent inclusion of this FG in higher DIAAS meals. In contrast, while nuts and seeds also had similar weight contributions when present in meals (Figure 2), the difference in their weight proportion was not statistically significant between higher and lower DIAAS meals when the prevalence was accounted for (Figure 3). These observations reflect comparable prevalence and within-meal weight proportions across DIAAS strata.

Relative FG proportions that characterize higher and lower-protein quality meals are presented for the top representative FGs in Supplementary Table 1. Proportions were expressed as ratios relative to a reference FG, which was set to 1.0, and other FGs were expressed relative to that baseline. For example, when legumes and pulses were set as the reference, on average, grains and pasta had a value of 0.45 in the higher DIAAS meals, as compared to 1.39 in the lower DIAAS meals. This indicated a higher ratio of legumes: grains in higher protein quality meals. When nuts and seeds were set as the reference, legumes still dominated in higher DIAAS meals, while grains rose relative to nuts and seeds in poorer DIAAS meals. Fruits and vegetables contributed smaller weight proportions than grains, legumes, nuts, and seeds in both DIAAS strata. However, their relative contributions were less extreme in higher DIAAS meals as compared to other FGs.

### 3.2 PCA and k-means clustering

**Figure 4:**
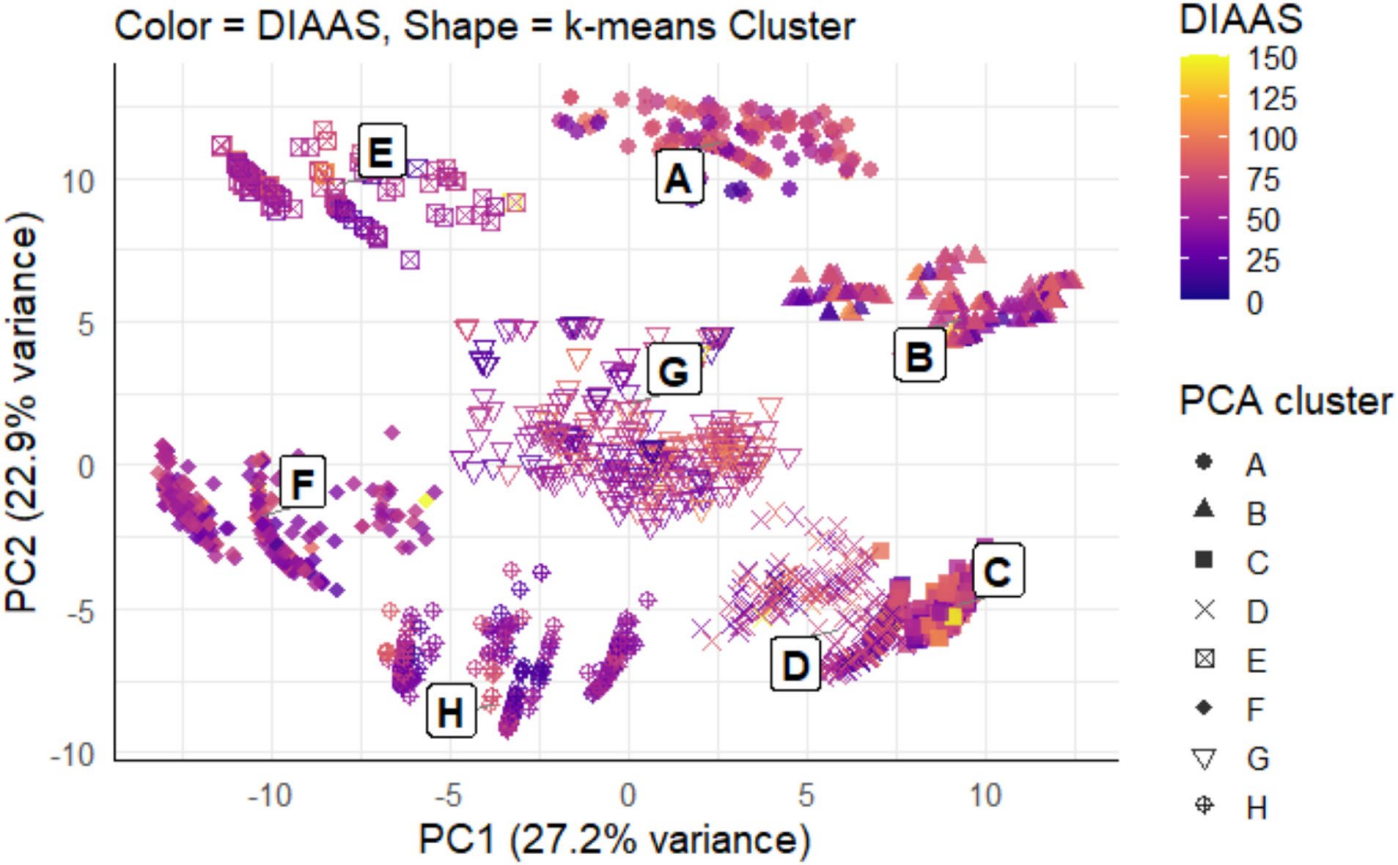
Cluster visualization of meal protein quality in a 2-dimensional plot, with shapes of plot points representing meals of different protein quality as distinguished by *k*-means clustering. The color of plot points represents the mean DIAAS of values, 10–150. Each cluster is labeled “A–H.” Labels were derived by calculating the centroid of each *k*-means cluster by taking the mean PC1 and PC2 values of all meals belonging to that cluster.

PCA and k-means clustering were data-driven techniques used to identify clusters of meal composition and how these clusters related to protein quality. Based on the silhouette analysis in Supplementary Figure 1, the optimal number of clusters was *k* = 6, where the mean silhouette width was the highest. However, the PCA plot showed more than 6 distinct clusters. To have a clearer distinction of meals with varied protein quality, *k* = 8 clusters, with a high mean silhouette width, were chosen and visualized in Figure 4. Cluster differentiation with *k* = 6 found convergence of cluster A with B and E, while C and D were combined. Overall, with approximately 50% of cumulative variance (as shown in PC1 and PC2 of Figure 4), the PCA resolved eight visually distinct meal types.

The loadings indicated the relative weight and direction of each FG along the axis (Table 3). The x-axis representing PC1 thus distinguished meals that were legume and vegetable-rich from those dominated by grain and nut-based foods. PC2, represented by the y-axis, was driven by positive loadings for nuts and seeds, fruit, legumes and pulses, isolates, and negative loadings for grains and pasta. Together, the two axes capture contrasts in meal structure within the dataset. The strongest differentiating factor in the data appears to be between meals dominated by legumes and pulses and those dominated by grains and pasta. Supplementary Figures 2a–h provide a two-dimensional binned density heatmap to visualize the contribution of each FG as meal proportions across the PCA score space.

**Table 3:** PC loadings of all FGs, indicating the relative weight and direction of each FG along the axis.

Food group	PC1 loading	PC2 loading
Fruits	−0.237	0.0834
Grains and pasta	−0.185	−0.852
Isolates	−0.0452	0.0587
Legumes and pulses	0.759	0.143
Nuts and seeds	−0.538	0.484
Potatoes, kumara, and taro	0.0548	0.0586
Vegetables	0.198	0.0641
Others (sugar and sweets, yeast and condiments, beverages)	−0.00717	−0.0405

Table 4 shows the mean DIAAS calculated for each of the eight clusters, which exhibited distinct FG compositions, some more aligned with higher protein quality. Clusters A to D, which had higher DIAAS, were positioned along the positive end of PC1, where legumes and pulses had the strongest loadings. Clusters E, F, and H, with the lower DIAAS, were positioned in the negative end of PC1, where fruit, nuts, and seeds, grains, and pasta load more strongly. Meals on the positive end of PC2, namely clusters A, B, and E, contain a larger share of nuts and seeds. In contrast, meals on the negative end of PC2 had strong loadings for grains and pasta.

**Table 4:** Mean DIAAS (%) and number of meals for each cluster.

Cluster	Mean DIAAS (SD)	Number of meals
A	67.7 (21.7)	133
B	67.6 (32.1)	558
C	67.4 (21.9)	175
D	63.1 (24.2)	423
E	62.8 (18.8)	430
F	56.3 (18.8)	362
G	54.6 (24.0)	506
H	47.7 (19.1)	694

Total number of meals = 3,281 as meals with DIAAS <10 were not represented in the PCA plot. sd = standard deviation.

Considering PC1 and PC2 jointly, variation in meal protein quality appears to be driven predominantly by legumes and pulses (strong positive on PC1), nuts and seeds (positive on PC2), and grains and pasta (negative on both PC1 and PC2). The remaining FGs show comparatively small absolute loadings, so they contribute less to discrimination among meals.

### 3.3 Association between meal types and daily clusters

Cross-tabulation of meal composition clusters A to H (derived from k-means on PCA scores) against daily dietary pattern clusters 1–3, as derived from dynamic time warping and hierarchical clustering (36) was conducted. This provided a probability distribution of meal protein quality clusters conditional on daily cluster membership: how likely is it that a certain meal composition is consumed by someone in a specific daily cluster? A similar probability analysis was repeated to test whether specific meal compositions were more common among individuals who exhibited no daily shortfalls in either protein or any IAA (37). Results are presented in Table 5.

**Table 5:** Conditional probability table showing relative frequency of each meal composition type (PCA clusters A to H) within (i) the three daily dietary clusters, based on temporal protein intake patterns across 24 h and (ii) groups of individuals with or without shortfalls in protein or at least one IAA (rows sum to 1).

Daily cluster	Presence of at least one shortfall	P_A	P_B	P_C	P_D	P_E	P_F	P_G	P_H
1	Yes	0.027	0.169	0.032	0.123	0.167	0.096	0.126	0.259
	No	0.031	0.177	0.046	0.116	0.174	0.085	0.158	0.212
2	Yes	0.043	0.134	0.068	0.134	0.082	0.136	0.170	0.233
	No	0.056	0.161	0.073	0.140	0.140	0.120	0.149	0.161
3	Yes	0.043	0.190	0.069	0.147	0.052	0.086	0.207	0.207
	No	0.053	0.252	0.050	0.119	0.109	0.106	0.194	0.117

“Yes” denotes the presence of a shortfall, and “no” indicates no shortfall. Probabilities (columns labeled “P_”) represent the likelihood that a meal type belonged to each daily cluster and whether there is a shortfall. Key probabilities for discussion are highlighted in red.

Daily clusters, as ranked by total protein intake and protein quality, were 3 > 2 > 1, with daily diets in cluster 3 exhibiting 3x higher protein and IAA intake per EO as compared to cluster 1. As described in Table 3, protein quality of meal types was in the order of DIAAS: A > B > C > D > E > F > G > H. Meal types A and B, characterized by the legume- and nut- dense foods showed the highest probability in daily clusters 2 and 3 and among individuals with no shortfalls (probabilities in red in columns P_A and P_B). In contrast, meal type H, characterized by grain-rich, legume- and nut-poor foods, was most strongly associated with daily cluster 1 and with individuals who had at least one shortfall (P_H, values in red). Interestingly, high-quality meal types were not exclusive to daily cluster 3, nor were low-quality meals confined to daily cluster 1, as we observed the presence of meal pattern H in clusters 2 and 3 as well, but more so for individuals with at least one shortfall (P_H = 0.233 for cluster 2 and 0.207 for cluster 3). Similarly, meals of higher protein quality (A, B, C) were also found in daily cluster 1, but at a higher probability for individuals with no shortfalls.

The chi-square test indicated a significant association between daily clusters and PCA-derived meal types [χ^2^ (14, *N* = 3,281) = 111.63, *p* < 0.001], suggesting that certain meal types were more common in specific daily dietary protein intake patterns. Similarly, a significant association was also obtained between meal types and the presence of at least one shortfall [χ^2^ (7, *N* = 3,281) = 43.3, *p* < 0.001]. Supplementary Table 2 shows the contingency table of PCA-derived meal types A to H vs. daily dietary clusters and the presence of at least one shortfall.

The adjusted Pearson residuals were scaled to be comparable across cells. Pearson residuals with values > + 2 indicate significant over-representation (observed > expected) and values < −2 showed significant under-representation (observed < expected) (38). As expected, meal types with lower DIAAS, such as E and H, were over-represented in daily cluster 1 (E: 3.81; H: 3.10), indicating that low protein quality meals occurred more frequently in this cluster than would be expected under independence (Supplementary Table 2). In contrast, these meals were underrepresented in cluster 3 (Pearson residual of −3.55 for meal H). Meals of higher protein quality, such as A, B, and C, were underrepresented in cluster 1 (A: −2.38; C: −2.93) but overrepresented in cluster 3 (B: 3.62). These similar patterns were also observed when comparing the distribution of meal types between individuals with and without daily shortfalls. To highlight, meal type H was overrepresented in individuals with at least one shortfall (Pearson residual of 3.35 in H) but underrepresented in individuals with no shortfalls (−4.15).

## 4 Discussion

### 4.1 Meal composition in meals of varying protein quality

Comparisons between meals with higher (DIAAS ≥75%) and lower protein quality (DIAAS <75%) found differences in FG compositions. While legumes and pulses, nuts and seeds, grains and pasta, and isolates were singled out as the primary food groups in vegan meals that contributed most to protein intake, their relative nutrient contribution differed between higher and lower protein quality meals (Figures 1, 2). Legumes were present in higher proportions to grains, starchy vegetables, fruits and sugary foods in higher protein quality meals and were thus key PB food sources to improve IAA content of meals, but more so for lysine, leucine, tryptophan, and threonine than for SAAs. Grains, nuts, and seeds appear to provide more SAAs and could be suggested as suitable food sources to complement legumes and elevate levels of IAAs within meals to be closer to reference values.

While the PCA did not use meal DIAAS in its construction, the mean DIAAS by cluster indicated a correspondence between legume and nut-dominant patterns and higher protein quality, while grain-dominated patterns showed lower protein quality (Figure 4). This is further supported by calculated weight proportions of the key protein-providing FG in each meal cluster. The largest proportions for legumes and pulses and nuts and seeds were observed in clusters A and B, with no contribution from grains. The proportion of legumes and pulses and nuts and seeds was at 1.0 in cluster B and E, respectively, while the proportion of grains was at 1.0 for cluster H. These results indicate the potential importance of considering appropriate food group ratios that combine to provide vegan meals of high protein quality.

Particular attention is often paid to the complementation of legumes and grain foods as they are most commonly integrated within plant-based meal contexts as staples. As shown in Supplementary Table 1, higher protein quality meals appear to have approximately a 2:1 ratio of legumes to grains, which led to more optimal IAA profiles. Similar to findings from the vProtein project, where different plant foods were combined in a simulation exercise, the optimal proportion for high protein quality was found in an exemplar of 60% soy to 25% rice (39). These patterns are also consistent with previous studies examining two- and three-PB combinations, where legumes often appear in greater quantities than grains to achieve higher usable protein and DIAAS (29, 40). In this study’s vegan meals of higher protein quality, nuts and seeds also appeared to be at higher quantities than grains, but not higher than legumes and pulses (Supplementary Table 1). However, our PCA results showed that meal cluster E, which was dominated by nuts and seeds, had a lower DIAAS compared to other meal clusters. This was because nuts and seeds consumed in this cluster were snacks and mostly consumed in smaller quantities in isolation rather than part of a mixed meal with other complementary foods. Other snack foods, like fruits and beverages made of isolates, were also prevalent in this cluster. This pattern may thus reduce the overall protein quality of the eating occasions in this cluster.

Protein quality can be adequate even in minimally diverse diets (41, 42), but as diets become increasingly plant-based, or in our case, where all animal-sourced foods are excluded, the proportions of legumes, grains, nuts, and seeds need to be critically examined (43). Even within the legume group, food items vary in their contribution to protein quality. For example, when vegan menus replaced chickpea with lentils, a higher protein quality was attained at a lower quantity of the replacement (44). In our vegan diaries, plant-based meat alternatives (PBMAs), often using pea or soy protein isolate as an ingredient, are food items in the legume and pulses food group that have high DIAAS. Processing mechanisms in the manufacture of PBMAs often reduce the quantities of ANFs and transform these foods to highly digestible sources of protein with amino acid profiles close to the reference values, albeit more limited in SAA (25, 45, 46). Yet, we also observe legumes and pulses such as soy products, lentils, black beans, and chickpeas being part of high DIAAS meals that contained no PBMAs. This demonstrates the value of traditional plant protein sources in the contribution to protein quality. Further verification with specific digestibility coefficients for different types of legumes and their proportions in mixed meals is a further area of research to uncover the impact on protein quality and micronutrient provision.

### 4.2 Protein quality at the meal and daily level

The cross-tabulation of meal composition clusters against daily dietary pattern clusters highlighted clear links between diets and meals. Meals of higher protein quality, characterized by higher legume to grain ratios (A, B, and C), were significantly overrepresented in daily cluster 3—the cluster with daily diets that had the highest mean protein and IAA intake per EO. Contrastingly, meals of lower protein quality, such as meal H, were significantly overrepresented in daily cluster 1, the cluster with daily diets that had the lowest mean protein intake and protein quality per EO. Cluster E, which was dominated by nuts and seeds, was also overrepresented in daily cluster 1. This coincides with a “grazing” or snack-like pattern of <10 g per EO that was observed in our previous study (36).

While there is an association between meal protein quality, daily cluster patterns, and the presence of shortfalls, meals with higher protein quality were not confined only to certain daily patterns, and this is conversely true for meals with lower protein quality. These findings underscore that daily dietary patterns do not always equate to consistent meal-level practices, necessitating the evaluation of protein quality at the meal level. The main reason driving research interest in meal-level protein distribution and protein quality lies in the postprandial rise in circulating IAAs, and more acutely for leucine following dietary protein ingestion, that directly impacts muscle protein synthesis (MPS) (47). Assessing only the daily total will obscure the evenness of protein and amino acid intakes and absorption across meals. This may have greater implications for metabolic function within certain demographics (e.g., the elderly) and those on long-term diets high in PB foods, which generally exert lower anabolic responses in skeletal muscle mass (48, 49).

The extent to which IAAs must be complemented at the meal or day level is still debated. Young and Pellet argued that complementation at each eating occasion is unnecessary, and IAA profiles can be complemented across the day to maintain net nitrogen balance, even for PB sources (50). However, the time window of this complementation remains unknown, with some evidence that the plasma IAA availability remains elevated for a shorter time period in PB proteins than animal-sourced proteins (51). This may be more relevant in vegan diets, where many foods have lower protein quality when consumed alone but achieve substantially higher protein quality when combined in the context of meals. Pending definitive evidence on the appropriate temporal window and the impact of PB mixtures on anabolic responses, recommending complementarity at the meal level is likely beneficial to improve daily adequacy.

In one controlled trial comparing isonitrogenous meals of complete, complementary, or incomplete protein profiles in adult women, there were no significant differences in postprandial and 24-h MPS rates across the meal types (51). However, the study found that greater response was elicited in complete (animal-based) and complementary (plant) sources than in the incomplete plant protein meal. The incomplete meal also resulted in higher glucose and insulin levels, showing that differences in protein quality at the meal level have an influence on metabolic outcomes beyond MPS. Collectively, this asserts that fully PB meals can elicit similar metabolic responses comparable to animal-based meals, with the caveat that AA complementarity must be ensured so that protein quality from the meal aligns closely with human requirements.

Taken together, these findings caution against relying solely on daily intake of protein and IAA intake (as explored in our past studies) as an estimation for protein adequacy, because this overlooks meal-level composition. Our approach to contextualize meal-level protein quality within overall dietary intake does not assume dependence between individual meals, as individuals will realistically consume different types of foods throughout the day. Considering that more than 50% of daily diets in the cohort had a shortfall in either protein or one of the IAAs, we aimed to understand which meal compositions tended to occur within broader daily dietary contexts. In the context of restrictive diets like the vegan diet, this analysis provided insight into whether individuals who consistently meet protein or IAA requirements tended to consume higher protein-quality meals across the day. We observe that meals that are composed mostly of legumes are more associated with daily diets with no shortfalls.

The majority of plant protein sources appear to come from grains, especially in dietary shifts toward predominantly PB diets (52, 53). PB diets characterized by poor diversity of PB foods therefore incur the risks of deficiencies in bioavailable IAAs, as well as other micronutrients. Complementarity of various PB protein sources consumed within limited time windows, such as at the meal level, is a means of overcoming such challenges (2).

The long-term physiological consequences of repeatedly consuming vegan meals with incomplete IAA profiles are unknown. If protein quality is not consistently ensured, there is a risk that the Recommended Dietary Intake—defined on the basis of high-quality reference proteins—may not be achieved in practice (6). In the NZ vegan population of this study, overall protein—even after adjustments for bioavailability—did not indicate a high risk of deficiency for the majority (>75%) of participants (13). However, lysine and leucine emerged as critical nutritional concerns, with approximately half of the cohort failing to meet the daily requirements. These findings emphasize the importance of analyzing meal composition for protein quality, a central aim of our study. By analyzing the composition and proportion of FGs in vegan meals, this study seeks to identify patterns that provide higher protein quality and, in turn, inform strategies to optimize meal planning for vegans to better meet individual nutrient requirements.

### 4.3 Strengths and limitations

All diets can incur nutrient shortfalls if food groups are poorly balanced. For restrictive dietary patterns, such as vegan diets, where nutrients are solely provided from fewer food groups, evidence-based research is needed to provide practical solutions to overcome nutritional limitations. A key contribution of this study was the use of meal-level analysis, which uncovered critical food groups such as legumes and isolates to achieve high protein quality in vegan meals. This study was limited by the foods that vegan participants reported consuming. While this provided a realistic snapshot of what NZ vegans typically eat, it does not capture the full range of all PB products, including more novel foods such as algae and mycoprotein, which may have nutritional potential as meat substitutes and contribute to higher protein quality (54). Our analyses reflect the PB combinations reported by the cohort, and these food pairings within each EO may not be generalized to other vegan populations. However, we make the case that the food diaries obtained are representative of a western-style vegan diet due to its similarity in patterns with other vegan populations in the West, where legumes, textured soy and other meat analogues, grains, nuts, and seeds dominate protein intake (55–57). Time interval analysis is an effective means of assessing IAA complementation within EOs (52). We defined a meal strictly as foods co-reported at the same timestamp, rather than using a proximity window. Given the lack of consensus on optimal inter-meal intervals for metabolic outcomes, we opted for the more certain time-stamp definition. However, this approach may miss foods eaten shortly before or after an EO that could have been part of the same meal and influence its protein quality.

Another limitation of this study is the reliance on incomplete or imprecise amino acid composition data and on broadly grouped TID values for many PB foods. Owing to current data paucity, individual food items were assigned the digestibility coefficients of the broader food group, which can overlook variation within food groups and a lack of granularity in calculating DIAAS. Data for certain IAAs (such as tryptophan) were absent for some foods, making the calculation of a correct DIAAS value impossible. Furthermore, the lack of TID adjustments for isoleucine, valine, and the aromatic AAs means that DIAAS calculations did not incorporate the protein quality measurements of these IAAs. These IAAs are, however, not commonly limited in PB foods. For example, valine is most limiting in pork and beef, while lysine is identified as most limiting for several PB foods (2, 46). Consequently, in the context of this study, where vegan diets are analyzed, the exclusion of TID-values for these IAAs may not impact protein quality assessments extensively, as protein quality is primarily constrained by lysine and SAAs in this study. Although isoleucine, valine, and the AAA intake were not adjusted for digestibility, these nutrients were noted to be above requirements in our previous study (13). Insufficient or inaccessible TID and AA profiles continue to hamper research in protein quality (58). Nevertheless, more detailed composition and digestibility data for PB foods are becoming available (59), so replacing current estimates with these values will allow for more precise conclusions in future studies. As each IAA was expressed per gram of protein, foods with inherently low protein density, such as vegetables, will appear artificially inflated in IAA contribution (16). However, these foods contributed minimally to protein quality in the vegan meals in this study and were often present as part of a larger meal or recipe.

Our study is limited by a lack of standardization on total protein intake. Interpretation of protein quality from our observational study reflecting habitual eating patterns of vegans may be confounded by energy consumption or energy under-reporting. This was identified in our previous study where under-reporters had significantly lower protein intakes than plausible reporters (0.56 vs. 0.87 g/kg/day, *p* < 0.05). Our findings will therefore benefit from verification from well-controlled clinical trials (e.g., isonitrogenous diets), although conclusions may still be based on short-term diets (60).

One strength of the study was the use of unsupervised data-driven techniques such as PCA and k-means clustering that were applied to real-world meal records and linked to a robust protein quality metric (DIAAS). This approach identified the food groups most strongly associated with protein quality in vegan meals and outlined possible ranges of food proportions that underpin higher protein quality meal patterns. This sets the foundation for the strategic complementation of various plant foods to improve the protein quality of vegan meals—the next phase of this research. The quantity needed to meet individual requirements should be considered in addition to the DIAAS, which only accounts for protein quality in 1 g of a protein source. This provides limited insight, as, without sufficient intake, even proteins that are completely utilizable will fall short of meeting IAA requirements (61). We aim to account for these aspects in future studies. Lastly, protein quality is only one area of concern in vegan diets. Several micronutrients are often lacking in these diets, especially if fortified foods and supplementation are absent (3, 62). While this is not the area of focus in this study, meal-level analysis of vegan diets should take into consideration the daily requirements of certain vitamins and minerals, as well as potential meal-level requirements if applicable.

## 5 Conclusion

Our analyses indicate that vegan meals with lower protein quality are characterized by higher contributions from fruit, grain foods, potatoes, and sugary condiments, whereas meals with greater proportions of legumes and pulses—often exceeding grains—and, to a lesser extent, nuts and seeds, are associated with higher DIAAS. However, conclusions from these composition analyses should be interpreted with caution. We have evaluated protein quality using a single metric (DIAAS), and our inferences are specific to the foods and proportions of these foods in our vegan cohort, rather than a universal reflection of other populations. Further studies should test a wider range of food combinations and ratios and relate meal compositions not just to IAA reference values, but also evaluate the implications for metabolic outcomes.

Comparing meal-level and daily patterns suggests that assessing protein adequacy only at the daily scale can overestimate true adequacy because within-day protein distribution and complementation are unknown. Individuals may achieve similar total daily protein intake yet consume meals of varied numbers and quality. This may have potential physiological implications. Evaluation of protein adequacy should thus account for both daily totals and meal quality. A practical implication is the need to provide approximate quantities of these foods in mixed meals so that realistic protein-adequate vegan meals can be constructed. In the next phase of our research, we intend to illustrate this with meal complementation and optimisation modeling in a digital tool to tailor mixed meals to individual requirements and preferences.

## Data Availability

The original contributions presented in the study are included in the article/Supplementary material, further inquiries can be directed to the corresponding author.
