# Supplementary figures and images for: Beyond daily totals: meal-level digestible indispensable amino acid score reveals how food groups shape protein quality in vegan diets
Source: Front Nutr. 2026 Feb 12;13:1752697. doi: 10.3389/fnut.2026.1752697 (PMC12935615; doi:10.3389/fnut.2026.1752697)

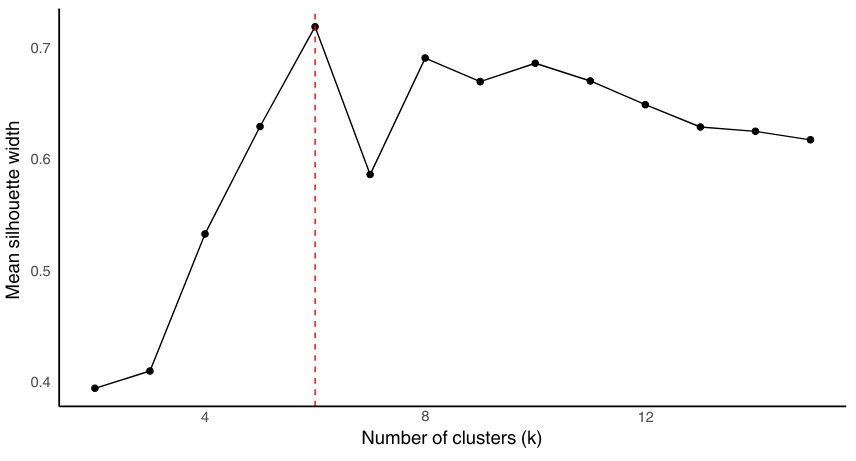

Supplement: Supplementary file 1 [file Image_1.tiff]
