# Supplementary material for: Beyond daily totals: meal-level digestible indispensable amino acid score reveals how food groups shape protein quality in vegan diets
Source: Front Nutr. 2026 Feb 12;13:1752697. doi: 10.3389/fnut.2026.1752697 (PMC12935615; doi:10.3389/fnut.2026.1752697)
Supplement: Supplementary file 2 [file Image_2.pdf]

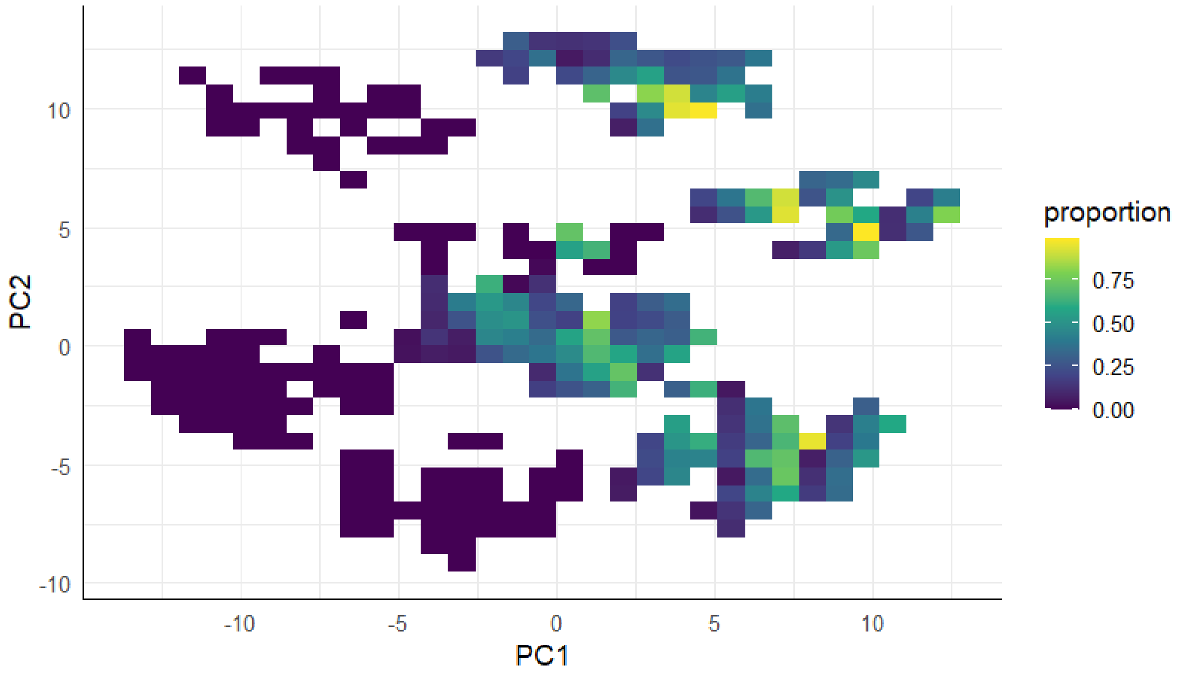

2a. Proportion of legumes across the PCA axes. Higher proportion of legumes within meal compositions were observed in the positive sides of PC1 and PC2.

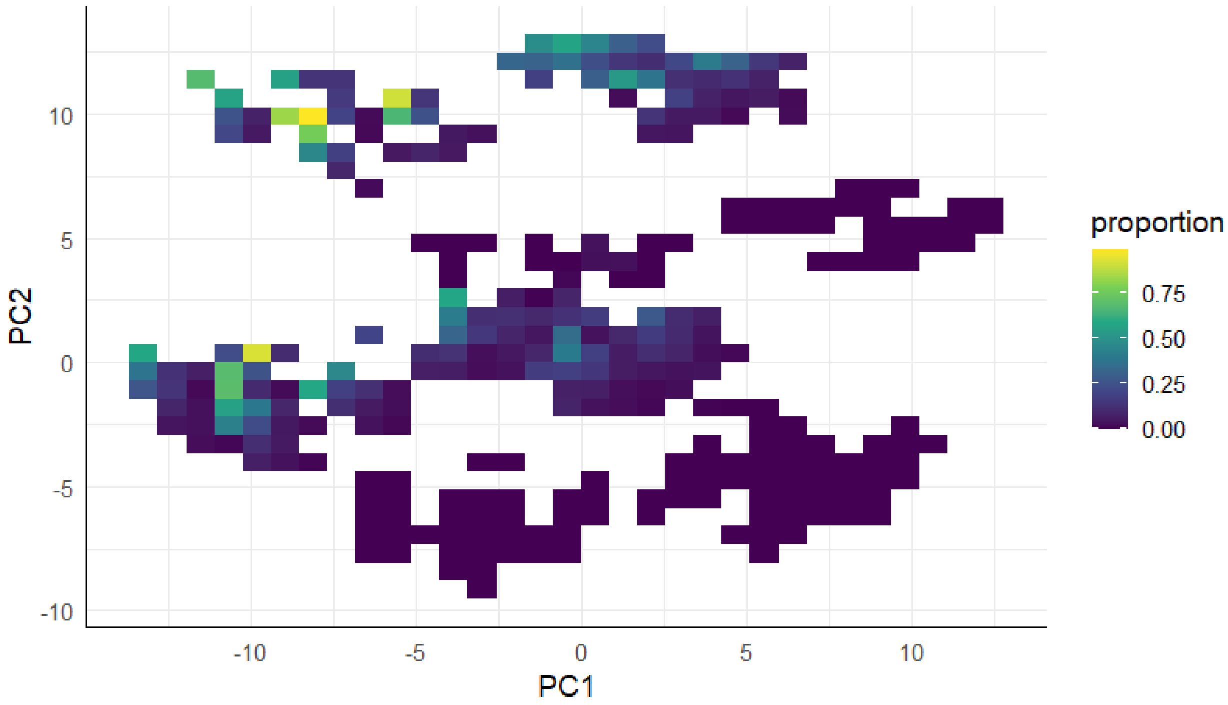

2b. Proportion of nuts and seeds across the PCA axes. Higher proportion of nuts and seeds within meal compositions were observed in the negative side of PC1 but positive side of PC2.

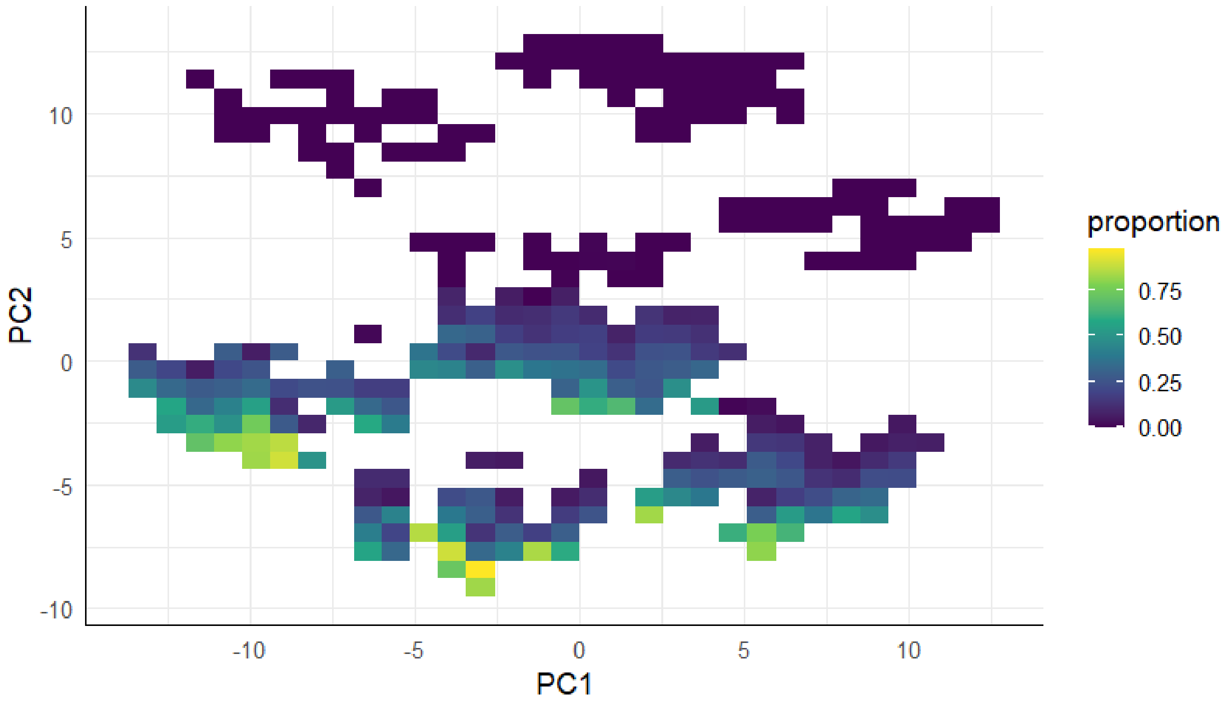

2c. Proportion of grains across the PCA axes. Higher proportion of grains within meal compositions were observed in the negative side of PC1 and PC2.

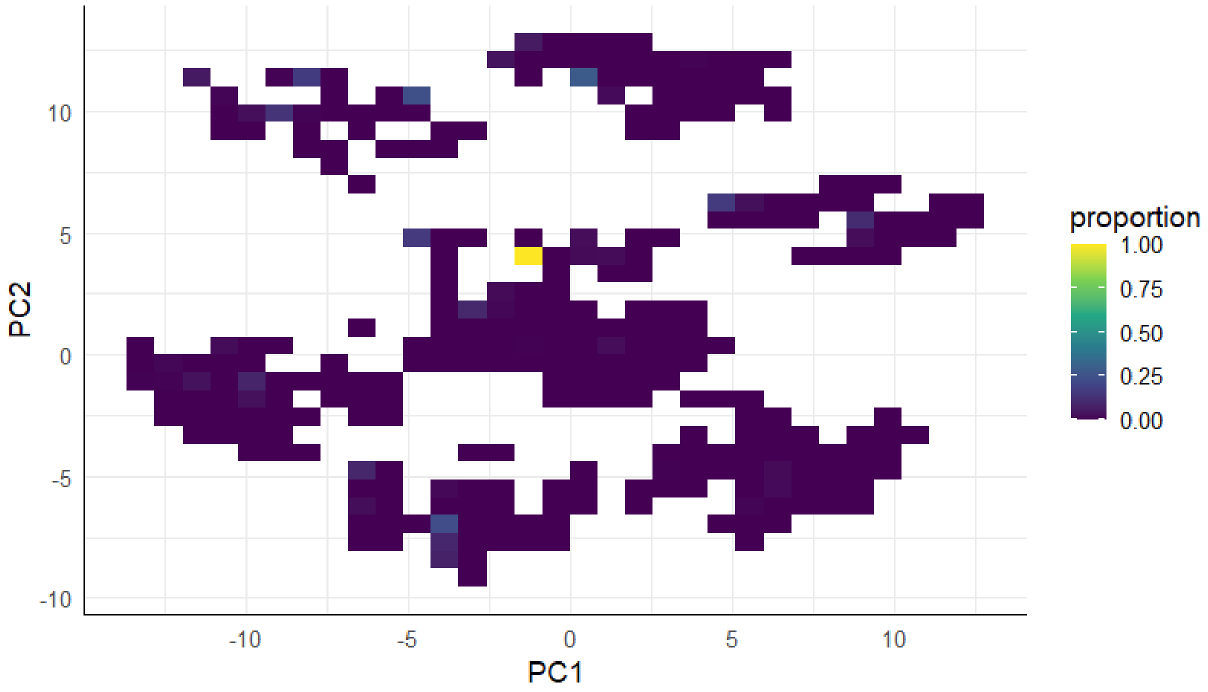

2d. Proportion of isolates across the PCA axes. Very low proportions were observed in all meal compositions and equally spread across the PCA axes, indicating isolates were not a dominant FG in all clusters.

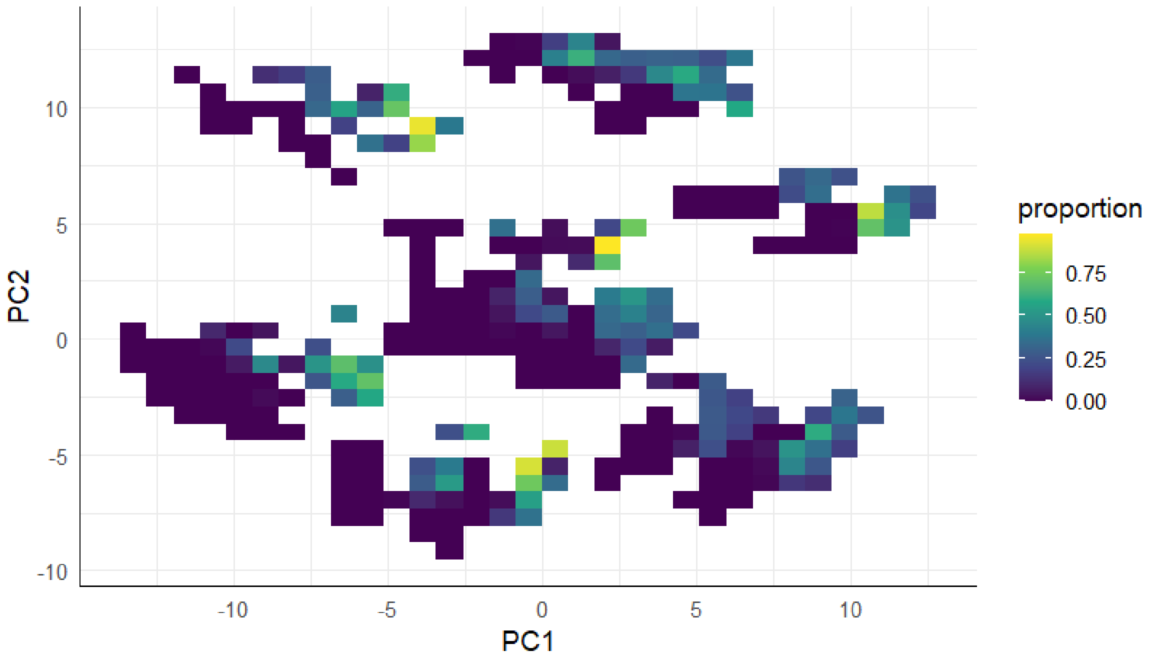

2e. Proportion of vegetables across the PCA axes. Relatively equal contribution of vegetables spread across all clusters, indicating substantial proportions in each meal.

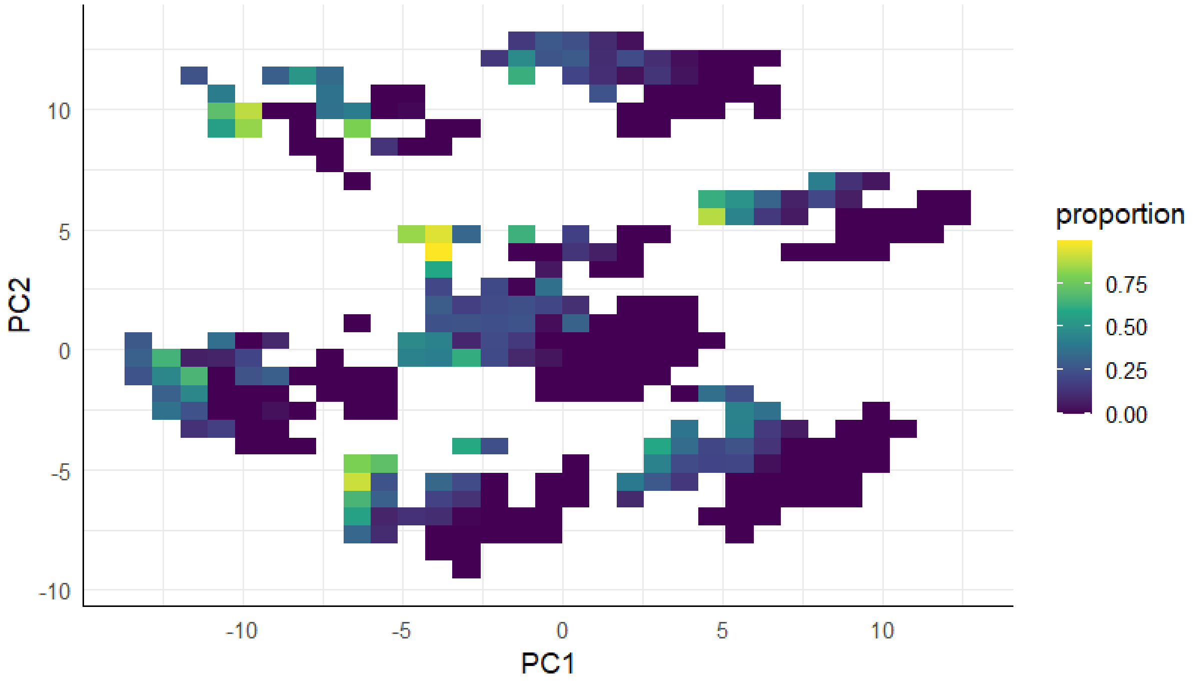

2f. Proportion of fruits across the PCA axes. All clusters were characterised by some proportions of fruits, but higher proportions were observed in meal compositions found in the negative side of PC1.

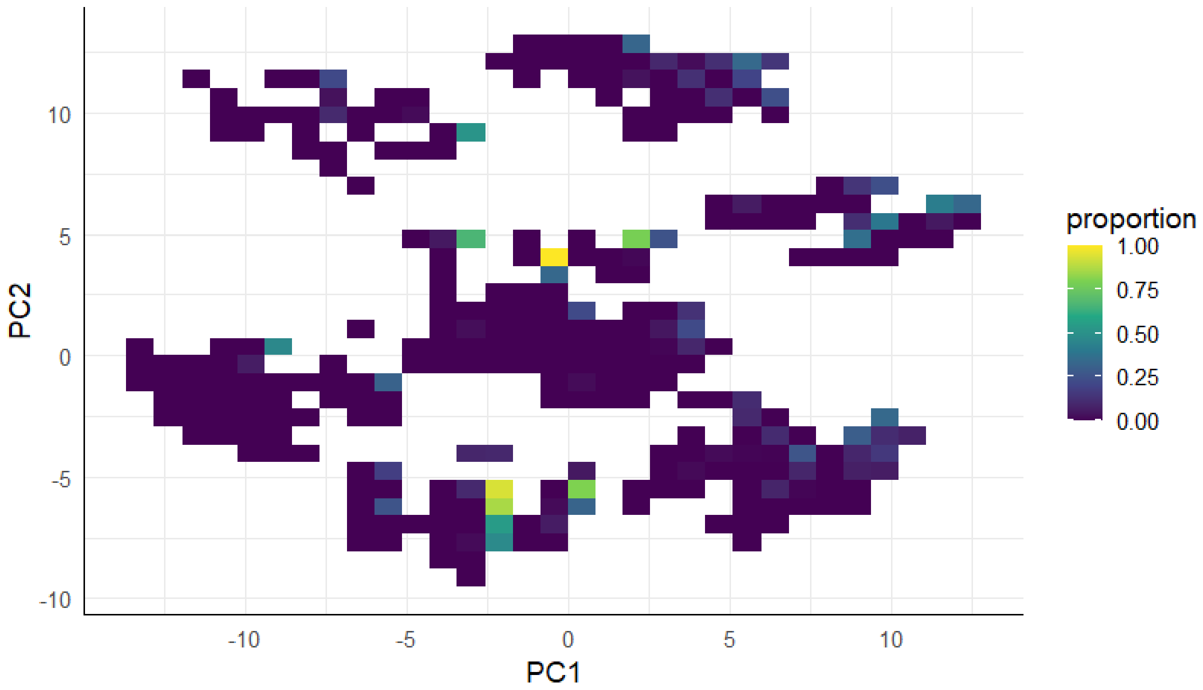

2g. Proportion of potatoes, kumara and taro across the PCA axes. Slightly higher proportion in meal compositions in the negative side of PC1 and PC2 but generally do not contribute highly to meals.

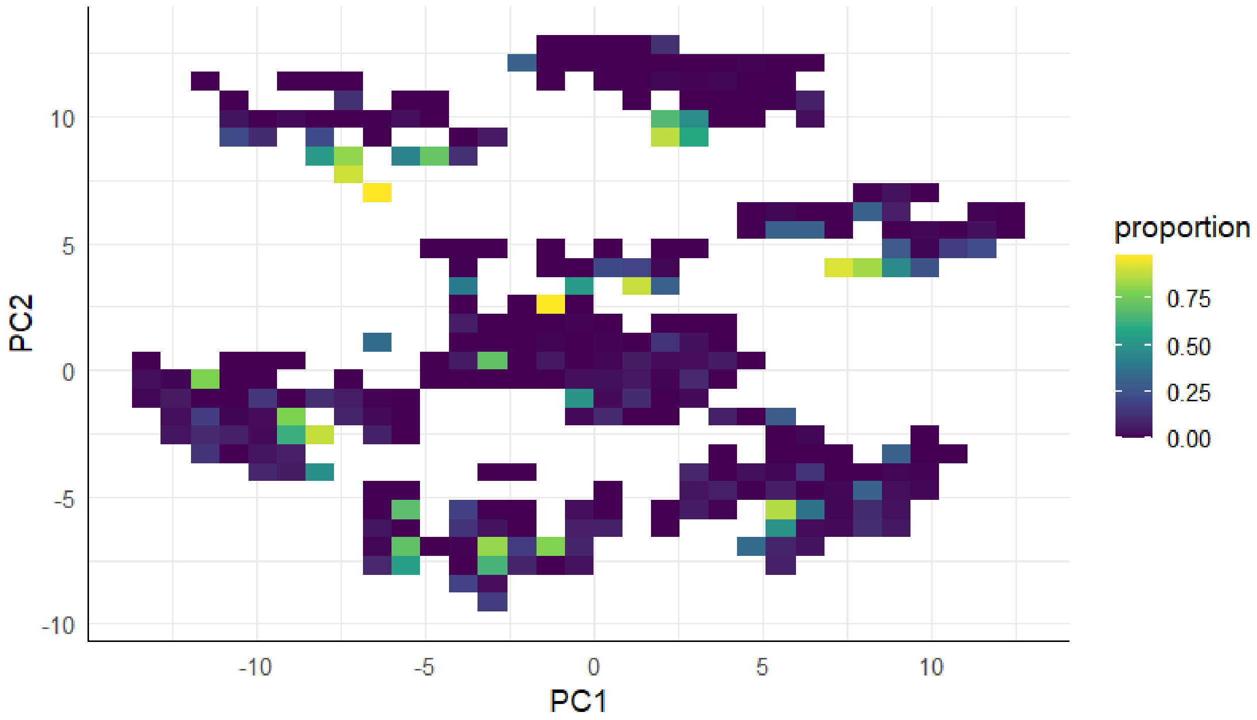

2h. Proportion of "others" comprising minor FGs, "beverages", "sugar and sweets" and "sauces and condiments". The distribution of these foods appears to be equal across all clusters but meal compositions aligned with higher protein quality in the positive spaces of PC1 and PC2 have lower proportions.

Supplementary Figure 2. Contribution of all FGs across meal composition patterns in a two-dimensional binned density heat map in a PCA score space that was divided into 30 by 30 bins. Lighter tiles show higher proportion of the FG in the meal composition. Distribution of FGs across the PCs were visualised for legumes and pulses **(A)**, nuts and seeds **(B)**, grains and pasta **(C)**, isolates **(D)**, vegetables **(E)**, fruits **(F)**, potatoes, kumara and taro **(G)** and other minor FGs **(H)**.
