# Supplementary material for: Beyond daily totals: meal-level digestible indispensable amino acid score reveals how food groups shape protein quality in vegan diets
Source: Front Nutr. 2026 Feb 12;13:1752697. doi: 10.3389/fnut.2026.1752697 (PMC12935615; doi:10.3389/fnut.2026.1752697)
Supplement: Supplementary file 3 [file Table_1.docx]

**Supplementary Table 1.** Relative food group ratios characterising higher and lower- protein quality vegan meals. Each row uses one food group as the reference ratio, set to 1.00, and values in the row indicate the proportional contribution of other food groups compared to that reference. For example, in higher DIAAS meals, when grains were set to 1.00, legumes appeared on average 2.205 times greater. Ratios for isolates are not shown in this table as other FGs have very elevated ratios (> 100) relative to isolates, possibly because isolates contribute comparatively lower weight in proportion to other foods.

|  | Meal protein quality based on DIAAS | | | | | | | | | |
| --- | --- | --- | --- | --- | --- | --- | --- | --- | --- | --- |
|  | Higher | Lower | Higher | Lower | Higher | Lower | Higher | Lower | Higher | Lower |
| FGs | Ratio to grains | | Ratio to legumes | | Ratio to nuts/seeds | | Ratio to fruits | | Ratio to vegetables | |
| Grains | 1.00 | 1.00 | 0.45 | 1.39 | 1.37 | 1.88 | 3.31 | 7.02 | 2.03 | 13.1 |
| Legumes | 2.20 | 0.72 | 1.00 | 1.00 | 3.03 | 1.36 | 7.29 | 5.06 | 4.47 | 9.41 |
| Nuts/seeds | 0.73 | 0.53 | 0.33 | 0.74 | 1.00 | 1.00 | 2.41 | 3.73 | 1.48 | 6.92 |
| Fruits | 0.30 | 0.14 | 0.14 | 0.20 | 0.42 | 0.27 | 1.00 | 1.00 | 0.61 | 1.86 |
| Vegetables | 0.49 | 0.08 | 0.22 | 0.11 | 0.68 | 0.14 | 1.63 | 0.54 | 1.00 | 1.00 |

**Supplementary Table 2**. Contingency table of daily clusters (1-3) x PCA meal composition clusters (A-H) and presence/absence of shortfall (Yes/No) x PCA meal composition clusters. Key Pearson residuals selected for discussion are highlighted in red.

| Daily cluster |  | A | B | C | D | E | F | G | H | Total |
| --- | --- | --- | --- | --- | --- | --- | --- | --- | --- | --- |
| Cluster 1 | Observed | 37 | 230 | 47 | 164 | 227 | 126 | 178 | 337 | 1346 |
|  | Expected | 54.6 | 228.9 | 71.8 | 173.5 | 176.4 | 148.5 | 207.6 | 284.7 | 1346 |
|  | Pearson residual | -2.38 | 0.07 | -2.93 | -0.72 | 3.81 | -1.85 | -2.05 | 3.10 |  |
|  | Adjusted Pearson residual | -3.16 | 0.10 | -3.92 | -1.00 | 5.32 | -2.55 | -2.91 | 4.54 |  |
| Cluster 2 | Observed | 71 | 211 | 101 | 197 | 156 | 186 | 231 | 289 | 1442 |
|  | Expected | 58.4 | 245.3 | 76.9 | 185.9 | 189.0 | 159.1 | 222.4 | 305.0 | 1442 |
|  | Pearson residual | 1.64 | -2.19 | 2.75 | 0.81 | -2.40 | 2.13 | 0.58 | -0.92 |  |
|  | Adjusted Pearson residual | 2.24 | -3.21 | 3.77 | 1.12 | -3.44 | 3.02 | 0.84 | -1.38 |  |
| Cluster 3 | Observed | 25 | 117 | 27 | 62 | 47 | 50 | 97 | 68 | 493 |
|  | Expected | 20.0 | 83.8 | 26.3 | 63.6 | 64.6 | 54.4 | 76.0 | 104.3 | 493 |
|  | Pearson residual | 1.12 | 3.62 | 0.14 | -0.20 | -2.19 | -0.60 | 2.40 | -3.55 |  |
|  | Adjusted Pearson residual | 1.24 | 4.31 | 0.15 | -0.23 | -2.55 | -0.69 | 2.84 | -4.34 |  |
| Presence of shortfall |  |  |  |  |  |  |  |  |  |  |
| Yes | Observed | 68 | 311 | 96 | 256 | 252 | 221 | 294 | 489 | 1987 |
|  | Expected | 80.5 | 338 | 106 | 256.1 | 260.4 | 219.2 | 306.4 | 420.3 | 1987 |
|  | Pearson residual | -1.40 | -1.46 | -0.97 | -0.01 | -0.52 | 0.12 | -0.71 | 3.35 |  |
|  | Adjusted Pearson residual | -2.27 | -2.56 | -1.59 | -0.02 | -0.89 | 0.20 | -1.23 | 6 |  |
| No | Observed | 65 | 247 | 79 | 167 | 178 | 141 | 212 | 205 | 1294 |
|  | Expected | 52.5 | 220 | 69 | 166.8 | 170 | 142.8 | 199.6 | 273.7 | 1294 |
|  | Pearson residual | 1.73 | 1.82 | 1.21 | 0.01 | 0.65 | -0.15 | 0.88 | -4.15 |  |
|  | Adjusted Pearson residual | 2.27 | 2.56 | 1.59 | 0.02 | 0.89 | -0.20 | 1.23 | -6 |  |

*Pearson value > 2 or 3 (highlighted in red) has a significant deviation from expectancy (30)
